# Artificial Intelligence-Based Histopathological Analysis to Assist Pathologists in Diagnosing Ewing Sarcoma and Selected Tumor Entities Using Tissue Microarrays

**DOI:** 10.3390/ijms27156864

**Published:** 2026-07-31

**Authors:** Francisco Giner, Álvaro Pastor-Naranjo, Pablo Meseguer, Rocío Del Amor, Marco Gambarotti, Alberto Righi, José Antonio López-Guerrero, Samuel Navarro, Empar Mayordomo-Aranda, Antonio Llombart-Bosch, Valery Naranjo, Isidro Machado

**Affiliations:** 1 Pathology Department, University of Valencia, 46010 Valencia, Spain; francisco.giner@uv.es (F.G.); samuel.navarro@uv.es (S.N.); empar.mayordomo@uv.es (E.M.-A.); antonio.llombart@uv.es (A.L.-B.); 2 Pathology Department, Hospital Universitari i Politècnic La Fe, 46026 Valencia, Spain; 3 Instituto Universitario de Investigación e Innovación en Tecnología Centrada en el Ser Humano (HUMAN-Tech), Universitat Politècnica de València (UPV), 46026 Valencia, Spain; apasnar@alumni.upv.es (Á.P.-N.); pabmees@upv.es (P.M.); madeam2@upvnet.upv.es (R.D.A.); vnaranjo@dcom.upv.es (V.N.); 4 Artikode Intelligence S.L., 46022 Valencia, Spain; 5 Department of Pathology, IRCCS Rizzoli Orthopaedic Institute, 40136 Bologna, Italy; marco.gambarotti@ior.it (M.G.); alberto.righi@ior.it (A.R.); 6 Molecular Biology Department, Instituto Valenciano de Oncología, 46009 Valencia, Spain; jalopez@fivo.org; 7 Department of Pathology, Catholic University of Valencia, 46001 Valencia, Spain; 8 Joint Cancer Research Unit, Centro de Investigación Príncipe Felipe (CIPF), 46024 Valencia, Spain; 9 Pathology Department, Hospital Clínic Universitari, 46010 Valencia, Spain; 10 Centro de Investigación Biomédica en Red de Cáncer (CIBERONC), 28029 Madrid, Spain; 11 Instituto de Investigación Sanitaria, Instituto de Investigación Sanitaria del Hospital Clínico Universitario de Valencia (INCLIVA), 46010 Valencia, Spain; 12 Pathology Department, Instituto Valenciano de Oncología, 46009 Valencia, Spain; 13 Patologika Laboratory Hospital Quirónsalud, 46010 Valencia, Spain

**Keywords:** Ewing sarcoma, round-cell sarcomas, artificial intelligence, image diagnoses

## Abstract

Ewing sarcoma is a highly malignant tumor whose histological appearance often overlaps with that of other undifferentiated round-cell and ovoid-cell tumors, making accurate diagnosis challenging. Selecting the most appropriate immunohistochemical and molecular tests is critical, particularly when only limited core biopsy material is available and tissue preservation for additional molecular or biomarker testing is required. Artificial intelligence (AI)-based histopathological image analysis is emerging as a promising diagnostic tool in oncology. This study evaluated the potential of AI-based histopathological analysis to assist pathologists in the morphologic classification of Ewing sarcoma and selected histological mimics using tissue microarrays (TMAs), rather than to identify or predict molecular alterations. We analyzed 1926 digitized histological cores, from 729 patients, assembled into 45 tissue microarrays. The dataset comprised 517 Ewing sarcomas (ESs), 367 rhabdomyosarcomas (RMSs), 187 chondrosarcomas (CHSs), 138 gastrointestinal stromal tumors (GISTs), and 124 synovial sarcomas (SSs). A weakly supervised multiple-instance learning (MIL) framework with transformer-based aggregation was developed using only core-level diagnostic labels. The model achieved classification accuracies of 97.1% for Ewing sarcoma, 80.0% for rhabdomyosarcoma, 85.7% for gastrointestinal stromal tumors, 80.0% for chondrosarcoma, and 76.0% for synovial sarcoma. The overall classification accuracy was 91.6%, with no misclassifications between Ewing sarcoma and rhabdomyosarcoma. These findings highlight the model’s robustness in distinguishing tumor entities that present a well-recognized diagnostic challenge in routine pathology. The AI algorithm demonstrated strong potential as a diagnostic adjunct for assisting pathologists in the morphologic classification of Ewing sarcoma and selected tumor entities based on histopathological features. Although these tumor entities are characterized by specific molecular alterations, the model was not designed to identify or predict molecular alterations directly. Instead, it supports clinical decision-making by recognizing morphologic patterns associated with diagnostically defined tumor entities. The integration of AI-based histopathological analysis with immunohistochemistry and contemporary molecular diagnostic techniques has the potential to improve diagnostic accuracy, optimize the use of ancillary testing, enhance our understanding of genotype–phenotype relationships, and ultimately support more effective patient management.

## 1. Introduction

Undifferentiated round-cell neoplasms, including Ewing sarcoma (ES), represent one of the greatest diagnostic challenges in pathology [1,2,3,4,5]. These tumors share highly similar cytomorphologic features, typically composed of small round to ovoid cells with scant cytoplasm and uniform nuclei [1,2,3,4,5]. However, specific molecular alterations, particularly *EWSR1/FUS::ETS* gene fusions, support the diagnosis of ES [1,2,3,4,5]. The marked morphologic overlap among entities with distinct prognostic and therapeutic implications complicates the distinction between carcinoma, lymphoma, melanoma, and other sarcoma mimics based solely on histological findings [4,5].

To confirm a morphologic diagnosis, pathologists frequently rely on extensive immunohistochemical and molecular studies to establish tumor lineage and identify specific genetic alterations [1,2,3,4,5]. However, this process is often limited by the small amount of tissue available in biopsy specimens, particularly core needle biopsies, where material may be rapidly exhausted after multiple staining procedures. In addition, obtaining a repeat biopsy can be challenging, especially when tumors are located in anatomically difficult or high-risk sites.

Histopathology directly reflects the tumor genotype, as genetic alterations produce phenotypic changes that can be detected under the microscope using standard hematoxylin and eosin (H&E) staining [6]. Histopathological analysis is internationally recognized as one of the richest sources of diagnostic information in pathology. A single tissue slide can contain over 10^7^ image pixels, and typically, multiple slides are available for each case [7,8]. In recent years, deep learning applied to histological slides has emerged as a major advancement in pathology and cancer diagnostics, offering the ability to extract complex visual patterns beyond human perception [7,8,9,10,11,12,13]. When applied to histological images, deep learning enables automated tumor classification and subtyping, opening new possibilities for supporting diagnostic decision-making. This approach is particularly valuable in cases with limited biopsy material, where algorithmic pre-screening can help prioritize the most informative immunohistochemical and molecular assays, thereby preserving tissue for additional analyses. To date, no studies have specifically evaluated the potential of deep learning applied to histological images for the diagnosis of undifferentiated round-cell sarcomas with specific molecular alterations, including Ewing sarcoma. The present study aimed to determine whether deep learning models trained on hematoxylin and eosin (H&E)-stained tissue microarray (TMA) core samples could accurately differentiate the major subtypes of round-cell sarcomas from their histological mimics, thereby improving diagnostic accuracy and optimizing tissue management. To achieve this, we implemented a weakly supervised multiple-instance learning (MIL) framework that leverages foundation models pretrained on large-scale histopathological datasets. This approach enables training using only slide-level diagnostic labels while preventing information degradation associated with image downsampling. By leveraging high-quality feature representations derived from domain-specific pretrained encoders, the model achieves accurate tumor classification in data-limited settings and supports the histopathological classification of morphologically distinct tumor entities, including Ewing sarcoma and other morphologically similar round-cell, ovoid-cell, and spindle-cell sarcomas.

## 2. Results

The classification performance for the core-level sarcoma subtyping task is reported in Table 1. The model achieves 93.6% and 81.6% accuracy scores on the development and test subsets, respectively. The balanced accuracy score that considers the class imbalances reaches 90.0% and 83.8%. The model achieves comparable performance between the internal validation and the test set, showing narrow confidence that demonstrates model consistency.

Given the inherent class imbalance of the dataset, we report in Table 2 the recall scores for each of the five classes in the subtyping challenge. Ewing sarcoma achieves the highest proficiency, with 97.5% and 97.1% sensitivity on the development and test subsets. The AI system achieve a recall score higher than 80% on the development set and only underperforms on the synovial sarcomas of the test set due to class imbalance.

**Figure 1:**
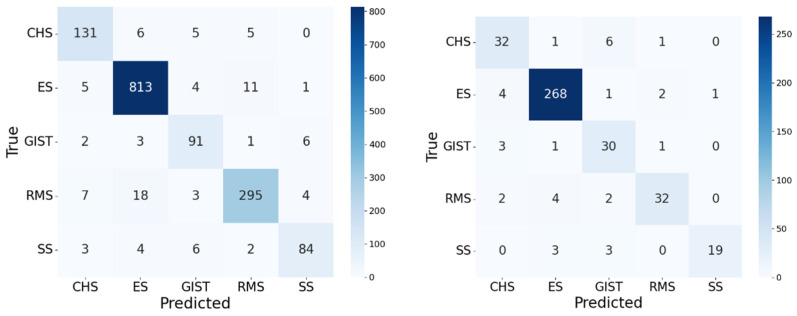
Confusion matrices of the sarcoma subtyping task using the proposed AY system. (**Left**) development set; (**right**) test set.

In Figure 1, we report the confusion matrix of the predictive model for the development and test cohorts. The system presents a marginal misclassification between ES and RMS, which represents the most challenging differential diagnosis between small round-cell tumors. The model achieves misclassification ratios between the two subtypes of 2.55% (29/1137) and 1.96% (6/306) for the development and test cohorts, respectively.

**Figure 2:**
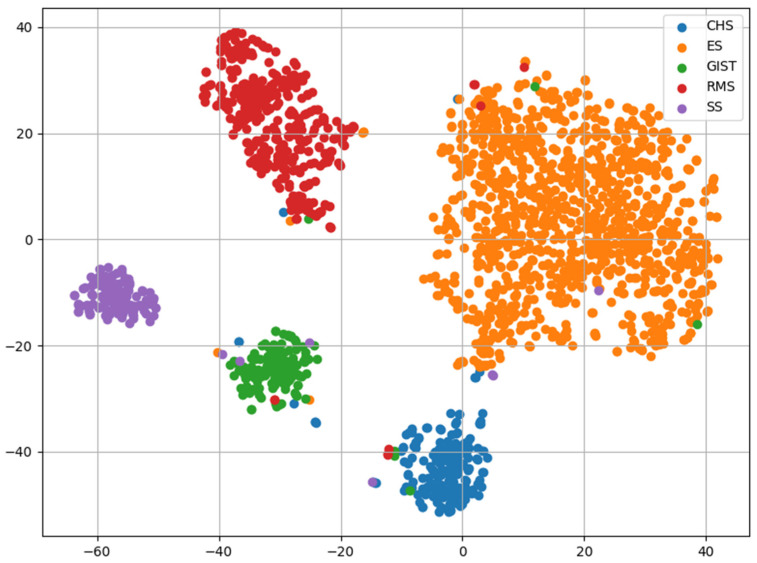
2D t-SNE representation of the learned core-level representation. Each point represents a single core and is colored according to the ground truth subtype.

Finally, we provide the 2D t-SNE projections of the learned core-level features in Figure 2. The proposed AI model demonstrated the extraction of meaningful representation from the images and the ability to create well-defined clusters for each class. These findings support the model’s proficiency in capturing disease-oriented patients from the histological images despite the notable morphological overlap of small round-cell tumors.

## 3. Discussion

Although certain genetic alterations are associated with specific sarcoma entities, many oncogenic drivers, including gene fusions, may occur across distinct tumor types. Accordingly, molecular findings should not be interpreted in isolation but rather integrated with morphologic, immunohistochemical, and clinical context [1,2,3,4,5]. This is particularly relevant for entities defined primarily by recurrent genetic alterations, in which a purely molecular approach may be misleading. Over the past few decades, numerous studies have demonstrated a close relationship between genetic alterations and specific morphologic phenotypes in sarcomas [1,2,3,4,5]. Moreover, certain genetic abnormalities reproducibly give rise to characteristic morphologic patterns that may serve as structural signatures of underlying molecular events [1,2,3,4,5].

The ability of artificial intelligence (AI) algorithms to classify histopathological sarcoma entities is of considerable clinical interest, as it may assist pathologists in prioritizing subsequent immunohistochemical and molecular testing while preserving limited formalin-fixed, paraffin-embedded (FFPE) tissue for future biomarker analyses and clinical trial-related testing. In this study, we applied a multiple-instance learning (MIL) framework to classify histopathological images of several sarcoma entities, including Ewing sarcoma, using digitized tissue microarrays. Our findings highlight the potential of foundation models pretrained on large-scale histopathological datasets to enhance domain-specific feature learning and enable efficient model adaptation. This approach may support the diagnostic workflow and optimize the use of limited biopsy material, particularly in core needle biopsies. In addition, in settings with limited access to molecular diagnostics, such algorithms may serve as decision-support tools to assist pathologists in prioritizing appropriate immunohistochemical panels and molecular tests, thereby supporting accurate diagnosis while optimizing the use of limited tissue samples.

As digital scanning technologies have become increasingly reliable, computational pathology has emerged as an integral component of modern diagnostic workflows, enabling efficient computer-assisted diagnosis and streamlining pathology practice [14,15,16,17]. AI-based decision-support tools can improve diagnostic efficiency and accuracy, ultimately contributing to enhanced patient care.

In this study, we present an AI-based approach for the classification of Ewing sarcoma using digitized H&E-stained slides. Our model achieved strong performance in distinguishing Ewing sarcoma from histological mimics across both training and independent holdout datasets. This task remains inherently challenging due to substantial morphologic overlap among tumor entities. To address this, we implemented a weakly supervised, attention-based MIL framework that eliminates the need for manual patch-level annotation, which is time-consuming, labor-intensive, and subject to interobserver variability [18,19,20].

Overall, the model represents a promising approach for the identification of Ewing sarcoma within an AI-assisted diagnostic framework. Its strong discriminatory performance among morphologically similar entities highlights its potential utility in routine diagnostic practice, particularly in the differential diagnosis of round-cell tumors.

Future work should focus on further validation and refinement of the model, including the incorporation of additional tumor entities and optimization across a broader diagnostic spectrum. Systematic and standardized data collection will be essential to enhance the model’s clinical robustness, particularly for emerging Ewing-like tumors, such as *CIC*, *BCOR*, *PATZ1*, and *NUT*-altered neoplasms, as well as other undifferentiated sarcomas, small-cell osteosarcoma, mesenchymal chondrosarcoma, lymphoma, poorly differentiated carcinoma, and neuroblastoma. We are currently collecting additional cases of Ewing sarcoma and Ewing-like tumors to support both internal and external validation.

The present study has several limitations. These include intratumoral heterogeneity, particularly in relation to the use of tissue microarray (TMA) samples, as well as the inherent difficulty of classifying round-, ovoid-, or spindle-cell sarcomas based solely on histopathological features. These factors represent important challenges for AI-based classification. However, the tissue microarray (TMA) cores used in this study (2–3 cores measuring 3 mm in diameter per tumor sample) are comparable in size to the limited core biopsy specimens routinely encountered in diagnostic practice, supporting the potential clinical applicability of our findings. This study represents an initial proof of concept in which we aimed to include a large number of sarcoma subtypes using TMAs as a preliminary step toward subsequent whole-slide image analysis. The use of TMAs also enabled the efficient inclusion of many tumor samples on a limited number of slides, thereby reducing the time and cost associated with slide preparation, digitization, and computational analysis. To address these limitations, further validation using a broader range of whole-slide images representing diverse tumor subtypes, together with immunohistochemical and molecular data, will be essential to improve diagnostic accuracy, robustness, and generalizability.

In conclusion, the integration of AI into digital pathology holds considerable promise for enhancing diagnostic precision and supporting clinical decision-making. An AI algorithm capable of reliably distinguishing Ewing sarcoma from its histological mimics could become a valuable adjunct in routine diagnostic practice, particularly by assisting pathologists in the morphologic classification of diagnostically challenging tumor entities. Although the primary objective of the present study was not to identify or predict molecular alterations, the proposed algorithm accurately classified histopathological tumor entities, several of which are characterized by recurrent molecular alterations, including Ewing sarcoma, rhabdomyosarcoma, and synovial sarcoma. By recognizing the morphological features of these entities, the algorithm could assist pathologists in selecting appropriate immunohistochemical panels and molecular tests during the diagnostic workup, thereby preserving valuable tissue for subsequent molecular and biomarker analyses. This is particularly important in routine practice, where limited biopsy material may otherwise be exhausted by multiple immunohistochemical stains that ultimately prove unnecessary. The integration of AI-based histopathological analysis with immunohistochemistry and contemporary molecular diagnostic techniques has the potential to improve diagnostic accuracy, optimize the use of ancillary testing and limited tissue samples, enhance our understanding of genotype–phenotype relationships, and ultimately support more effective patient management and follow-up.

## 4. Material and Methods

### 4.1. Dataset

(a) Case Selection and Inclusion Criteria

Histological slides from genetically confirmed undifferentiated round-cell sarcomas and histological mimics were retrospectively collected from four hospitals in Spain and Italy over a 30-year period. Eligible tumors included Ewing sarcoma (ES), rhabdomyosarcoma (RMS), synovial sarcoma (SS), chondrosarcoma (CHS), angiosarcoma (AS), and gastrointestinal stromal tumors (GISTs). The inclusion criteria required tumors to exhibit a predominant round, ovoid, or sarcoma-mimicking morphology and to have a definitive diagnosis established by conventional histopathology, immunohistochemistry, and/or molecular techniques. Slides with extensive necrosis, fibrosis, or technical artifacts that compromised histological evaluation were excluded. For each case, a representative formalin-fixed, paraffin-embedded (FFPE) tissue section from core biopsy samples assembled in tissue microarrays (TMAs) and stained with hematoxylin and eosin (H&E) was selected. A single anonymized TMA slide was generated per case and labeled with a consecutive identification code linked to a secure spreadsheet accessible only to the principal investigator. The final dataset (Table 3) comprised 1926 digitized histological cores from 729 patients assembled into 45 tissue microarrays (TMAs). The dataset included 517 Ewing sarcomas (ES), 367 rhabdomyosarcomas (RMS), 187 chondrosarcomas (CHS), 138 gastrointestinal stromal tumors (GIST), and 124 synovial sarcomas (SS). Clinical, radiological, and follow-up data were not included in the analysis. The study was approved by the Institutional Review Boards of the participating institutions (CE-AVEC 636/2025/Tess/IOR and CEIM-FIVO 2025-01/Code DIGITSRCT2024).

(b) Slide Digitization

All selected H&E-stained TMA slides were digitized at 40× magnification using a 3DHISTECH scanner. The resulting images were anonymized and securely transferred to the computational pathology team for preprocessing and subsequent computational analysis.

**Figure 3:**
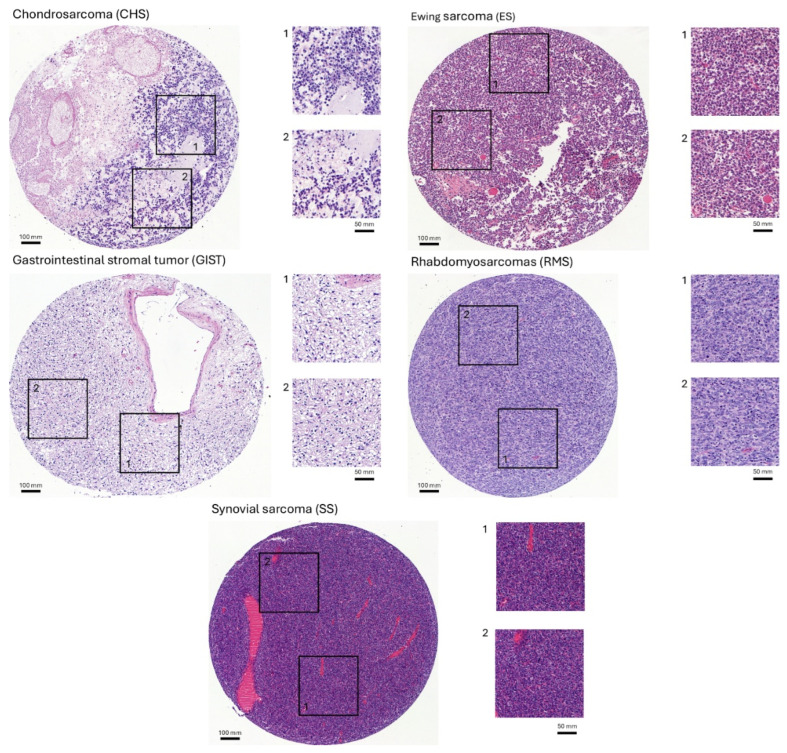
Some different selected biopsy cores of CHS, ES, GIST, RMS and SS in TMAs.

As shown in Figure 3, representative digitized biopsy cores of CHS, ES, GIST, RMS and SS illustrate the typical histomorphologic variability captured during the digitization process.

(c) Preprocessing

Each digitized TMA slide contained multiple cylindrical tissue cores, which were automatically detected and extracted to obtain individual tumor regions for analysis, typically measuring approximately 3000 × 3000 pixels. To enable efficient processing of these high-resolution images, each core was divided into non-overlapping image patches of 256 × 256 pixels. Tissue detection was performed using Otsu thresholding on the magenta channel, and patches containing less than 20% tissue were excluded. This preprocessing step reduced the computational burden while preserving relevant local histological patterns. Because only core-level diagnostic labels were available, the resulting patch-based representation naturally motivated the use of a weakly supervised multiple-instance learning (MIL) framework for subsequent analysis.

### 4.2. Methodology

#### AI Framework

(a) Weakly Supervised Learning

As described above, histological cores were divided into hundreds of small image patches, whereas only core-level diagnostic labels were available. In this setting, assigning labels to individual patches is impractical because manual annotation of every region by pathologists is highly time-consuming. This configuration defines a weakly supervised learning paradigm, in which the model learns global diagnostic patterns from partially labeled data. Weak supervision enables the network to identify the image regions that are most relevant for diagnosis without requiring exhaustive manual annotation, representing a key advantage in digital pathology, where expert annotation is both time-consuming and resource-intensive [21].

(b) Multiple-Instance Learning (MIL)

**Figure 4:**
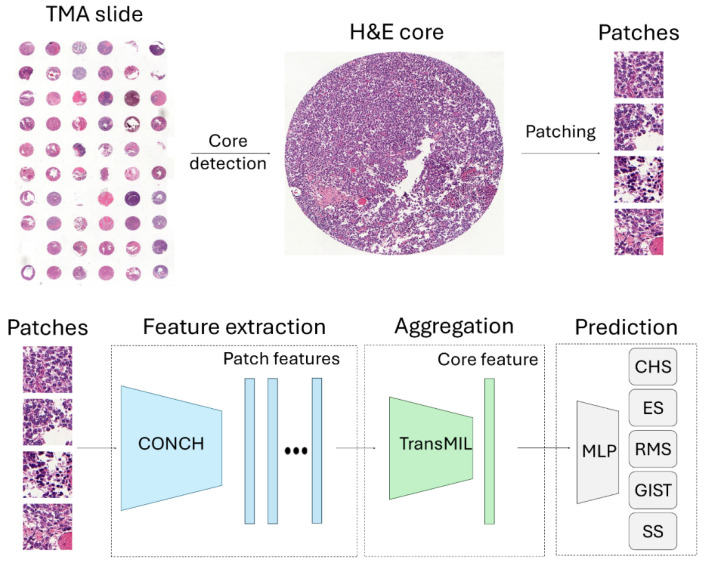
Overview of the multiple-instance learning (MIL) workflow, in which each TMA core image is represented as a bag of patches that are encoded and aggregated to generate a slide-level prediction.

To address this challenge, we adopted a multiple-instance learning (MIL) approach [22,23,24]. In MIL, each core is represented as a bag containing multiple instances (i.e., individual image patches). Each bag is assigned a single label corresponding to the core diagnosis, whereas the individual instances remain unlabeled (Figure 4). The model therefore learns to identify the instances within each bag that are most informative for predicting the core-level diagnosis.

In practical terms, each image patch was first encoded into a feature vector using a pretrained visual encoder. These feature vectors were then aggregated using a dedicated multiple-instance learning (MIL) architecture, which combined them into a single representation of the entire core [22,23,24]. By learning to assign adaptive weights to each patch according to its diagnostic relevance, the model was able to accurately classify complex histological patterns under weakly supervised conditions [22,23,24].

### 4.3. Feature Extraction with Foundation Models

In the multiple-instance learning (MIL) framework, model performance depends critically on the quality of the feature representations extracted from each image patch. To this end, we employed visual encoders based on pathology foundation models [25], which are large-scale neural networks pretrained on millions of images. Among these, models such as CONCH [26] have been further pretrained on histopathological datasets, enabling them to learn domain-specific representations of tissue morphology. This histology-specific pretraining allows the feature extractor to remain frozen during model training, while task-specific learning is performed exclusively by the MIL aggregator and classifier. Owing to the high quality of the extracted feature representations, this strategy facilitates effective adaptation to the target task of sarcoma subtype classification, even when only a limited number of training images are available.

Based on these considerations, we selected CONCH, one of the most widely used histopathology-specific foundation models available at the time of the study. Using this model, each image patch was encoded into a high-dimensional feature vector capturing both cytologic and stromal characteristics.

### 4.4. Feature Aggregation and Classification

After feature extraction, the patch embeddings from each core were aggregated using a multiple-instance learning (MIL) model, which combines information from all instances to generate a single core-level representation. This aggregation step enables the network to capture relationships among patches and integrate local and global histological features into a unified representation. For this purpose, we employed TransMIL [27], a transformer-based architecture specifically designed for MIL tasks. Through self-attention mechanisms, TransMIL models the spatial and contextual relationships among patches, enabling the network to assign greater weight to diagnostically relevant regions while preserving the overall tissue architecture. The aggregated feature vector was then passed to a multilayer perceptron (MLP) classifier, which generated the final diagnostic prediction corresponding to one of the five tumor types.

### 4.5. Experimental Settings

The dataset was divided into development (75%) and test (25%) subsets (Table 4), ensuring that all samples from a given patient were assigned to the same partition to prevent data leakage. The development subset was further partitioned into five folds for cross-validation, enabling internal validation and hyperparameter tuning. Performance metrics for the development set were calculated from the aggregated predictions across all cross-validation folds.

Models were trained using a weakly supervised multiple-instance learning framework with categorical cross-entropy as the loss function. Training was performed for 30 epochs for each hyperparameter configuration tested. The final model was optimized using the AdamW optimizer with a learning rate of 1 × 10^−5^ and a weight decay of 1 × 10^−5^. Performance was evaluated at the cohort level using accuracy (ACC), balanced accuracy (BACC), F1 score (F1S), and the area under the receiver operating characteristic curve (AUC). Class-level performance was assessed using recall. Ninety-five percent confidence intervals (CIs) were calculated using 1000 bootstrap resamples. Confusion matrices were used to visualize interclass misclassifications, and t-distributed stochastic neighbor embedding (t-SNE) [14,28] projections of the feature representations learned by the model for each core provided a qualitative assessment of the learned feature space.

#### Hardware and Software

Experiments were conducted on NVIDIA A100 GPUs with 40 GB of memory, enabling the training of large-scale deep learning models. Model development was performed in Python 3.10 using PyTorch (version 2.1.0), an open-source deep learning framework that provides modular layers, activation functions, and optimization algorithms for efficient neural network implementation. Image preprocessing was performed in MATLAB R2025b (MathWorks) because of its efficient matrix computation capabilities for large-format images. A Synology DS918+ network-attached storage (NAS) server with 32 TB of storage was used for data management and storage. The datasets and source code were hosted on the NAS and accessed remotely by the computer cluster through Secure Shell (SSH) tunnels established using MobaXterm v22.0, enabling the secure execution of training and inference workflows on the high-performance computing infrastructure.

## Figures and Tables

**Table 1:** Cohort-level figures of merit for the subtyping task on the development and test cohorts. Bootstrapped 95% CIs are reported.

	Development	Test
ACC	0.936 (0.924, 0.948)	0.916 (0.889, 0.940)
BACC	0.900 (0.876, 0.922)	0.838 (0.781, 0.893)
F1S	0.900 (0.878, 0.920)	0.845 (0.793, 0.892)
AUC	0.993 (0.990, 0.995)	0.987 (0.980, 0.993)

**Table 2:** Class-level recall for the subtyping task on the development and test cohorts. Bootstrapped 95% CIs are reported.

Class	Development	Test
CHS	0.891 (0.839, 0.935)	0.800 (0.677, 0.909)
ES	0.975 (0.964, 0.985)	0.971 (0.949, 0.989)
GIST	0.884 (0.813, 0.941)	0.857 (0.739, 0.966)
RMS	0.902 (0.870, 0.932)	0.800 (0.667, 0.928)
SS	0.848 (0.775, 0.918)	0.760 (0.576, 0.917)

**Table 3:** Class-level dataset counts regarding number of H&E cores, patients and tissue microarrays (TMAs). Overall number of patches extracted from the cores of each tumor subtype.

Subtype	Cores	Patients	TMAs	Patches
CHS	187	34	6	21,546
ES	1110	517	25	120,527
GIST	138	95	4	16,182
RMS	367	15	6	45,725
SS	124	68	4	23,402
Total	1926	729	45	227,382

**Table 4:** Class-level dataset counts for the development and test datasets. The number of H&E cores and patients (between parentheses) are reported for each subset and class.

Subset	CHS	ES	GIST	RMS	SS	ALL
Develop.	134 (26)	850 (388)	101(71)	230 (11)	92 (51)	1407 (547)
Test	53 (8)	260 (129)	37 (24)	137 (4)	32 (17)	519 (182)
Total	187 (34)	1110 (517)	138 (95)	367 (15)	124 (68)	1926 (729)

## Data Availability

The original contributions presented in this study are included in the article. Further inquiries can be directed to the corresponding author.
